# Differential Associations Between Parents' Versus Children's Perceptions of Parental Socialization Goals and Chinese Adolescent Depressive Symptoms

**DOI:** 10.3389/fpsyg.2021.681940

**Published:** 2021-06-25

**Authors:** Zexi Zhou, Mengtong Li, Jiawen Wu, Xiaoru Li

**Affiliations:** ^1^Department of Psychology, Fudan University, Shanghai, China; ^2^Department of Psychology, University of Illinois at Urbana-Champaign, Champaign, IL, United States

**Keywords:** adolescents, socialization goals, autonomy support, psychological control, depressive symptoms

## Abstract

Although prior studies have demonstrated the associations between parental socialization goals and parenting practices, as well as parenting practices and adolescent depressive symptoms, respectively, research examining the comprehensive developmental pathways among these constructs (i. e., the path from parental socialization goals to parenting practices to adolescent depressive symptoms) is scarce, especially in the Chinese context. Grounded in the integrative model of parenting, this study investigated the associations between parental socialization goals and adolescent depressive symptoms by examining the indirect pathways through parents' autonomy support and psychological control as well as the moderating effect of educational stage. In Study 1, 345 Chinese adolescents and their primary caregivers completed a measure on parental socialization goals. Adolescents also reported on their depressive symptoms. Results showed that children who reported more self-development parental goals showed fewer depressive symptoms. However, parents' reports of goals or child-parent perceptual discrepancies were not related to children's depressive symptoms. Drawing on this finding, 424 middle school and 301 high school Chinese adolescents completed measures regarding parental socialization goals, autonomy support, psychological control, and their own depressive symptoms in Study 2. Results showed that parental autonomy support linked the associations of self-development and achievement-oriented parental goals and children's depressive symptoms among middle school students, whereas parental psychological control linked such associations among high school students. Our findings provide a more holistic view on how parents' socialization goals are related to children's depressive symptoms via their parenting practices. We also discussed the practical implications for the clinical work regarding adolescent depressive symptoms.

## Introduction

The prevalence of depression rises sharply during adolescence, from <1% among children (Kessler et al., 2001) to 8–20% among youth (Naicker et al., 2013). A similar pattern of substantial increase is also shown regarding depressive symptoms not meeting the clinical diagnostic criteria of depression (Angold et al., 2002; Ge et al., 2006). The pooled prevalence of depressive symptoms was 19.85% among Chinese adolescents according to a recent meta-analysis (Rao et al., 2019). Adolescents struggling with depression are faced not only with considerable present challenges such as poor health condition and academic achievement (Prager, 2009; Thapar et al., 2012), but also with subsequent negative outcomes in later life, such as work impairment, higher risk of substance abuse, and intimate partner violence victimization (Miller et al., 2007; Naicker et al., 2013; McLeod et al., 2016). Therefore, identifying the risk and protective factors of adolescent depression and depressive symptoms is of great significance for their prevention and intervention.

Parental socialization goals refer to parents' values regarding the specific skills and general qualities they expect their children to acquire during socialization (Darling and Steinberg, 1993), which has a highly culture-specific feature shaped by distinct social values and ideal role models in different cultures (Bornstein and Cheah, 2006; Tamis-LeMonda et al., 2008). The integrative model of parenting suggests that parental socialization goals could influence parenting styles and parenting practices, which in turn affect adolescent developmental outcomes (Darling and Steinberg, 1993). However, although prior studies have demonstrated the associations between parental socialization goals and parenting practices (e.g., Rao et al., 2003; Luo et al., 2013; Chen-Bouck et al., 2019), as well as parenting practices and adolescent depressive symptoms (for a review, see Gorostiaga et al., 2019), respectively, research investigating the full process from parental socialization goals to adolescent depressive symptoms via parenting practices is scarce, especially in the Chinese context. Moreover, divergent findings on the effect of parental socialization goals on children's depressive symptoms were yielded when parental goals were reported by parents versus children. To address these concerns, this study aimed to investigate four research questions using Chinese samples: (a) whether there are discrepancies in child-reported and parent-reported parental socialization goals; (b) whether their respective perceptions of parental goals have differential associations with adolescent depressive symptoms; (c) whether parental autonomy support and psychological control link the significant relations between parental goals and adolescent depressive symptoms through indirect pathways; and (d) whether these relations vary across educational stages (i.e., middle school vs. high school).

### Parental Socialization Goals and Adolescent Depressive Symptoms

Three dimensions of parental long-term socialization goals toward adolescents in the Chinese context were proposed by Luebbe et al. (2018) based on the empirical evidence in line with prior theoretical works. First, self-development goals emphasize self-exploring and developing uniquely along with engaging with the broader world. Second, interdependence-oriented goals emphasize developing the awareness and ability to maintain interpersonal harmony and connection. Third, achievement-oriented goals emphasize attaining academic success and practicing filial piety (i.e., fulfilling family obligation). Only few studies have investigated the associations between these parental socialization goals and children's depressive symptoms, and notably, divergent results emerge in different studies. Specifically, Chinese children's report of maternal self-development goals was positively associated with their perceived maternal authoritative parenting, which was then negatively associated with their depressive symptoms (Li et al., 2010). However, Chinese mothers' report of their self-worth goals, which focused on enhancing children's self-regard so as to share conceptual similarities with self-development goals, did not predict children's emotional distress over time either directly or indirectly through parenting (Ng et al., 2019). A similar contradictory result was also observed in research regarding filial piety goals, which refers to parental expectation for their children to take family responsibilities such as respecting their elders and honoring the family. Chinese children's self-reported maternal filial piety goals were related to more depressive symptoms (Li et al., 2010), whereas Indian mothers' report of filial piety goals (i.e., relational goals) were unrelated to either teacher-reported children's adaptive functioning including happiness or child-reported sadness and anger management (Raval et al., 2014). With regard to the interdependence-oriented goals, only one study (Li et al., 2010) revealed the negative association between Chinese maternal collectivism goals reported by children and children's depressive symptoms.

The findings above suggest that different types of parental socialization goals held by parents may have divergent effects on children's depressive symptoms, but the inconsistent results regarding the same type of goals across studies may be due to the different informants completing the measures. Indeed, child-reports of self-development and filial piety goals were shown to be more related to their depressive symptoms compared with parent-reports of goals, which was consistent with prior works concerning parent-child perceptual discrepancies. Specifically, when evaluating parenting practices and children's adjustment (e.g., internalizing and externalizing problems), children's and parents' reports on the same variables often present no, or only weak to moderate correlations (De Los Reyes and Kazdin, 2005; Reidler and Swenson, 2012; Korelitz and Garber, 2016). Their respective views on parenting may further have differential predictive effects on children's depressive symptoms—typically, child-report has greater predictive power compared with parent-report (Reidler and Swenson, 2012; Laird and De Los Reyes, 2013; Human et al., 2016). Moreover, the parent-adolescent perceptual discrepancies in parenting were also found to be associated with adolescents' outcomes in some studies (De Los Reyes et al., 2010; Reynolds et al., 2011). For example, Fleming et al.'s (2015) findings suggested that child-reported more positive family management predicted their lower substance use likelihood when parents reported more positive family management synchronously. Such protective effect of child-report, however, disappeared when parents reported less positive family management. No research has tested whether such discrepancies exist concerning parents' and children's perceptions in parental socialization goals and their associations with adolescent depressive symptoms, which if exist, could be a possible explanation for the inconsistent results regarding the associations between parents' goals and children's depressive symptoms found in previous studies.

### Indirect Effects Through Parental Autonomy Support and Psychological Control

Although the relationships between parental socialization goals, parenting, and adolescent depressive symptoms have been initially explored in prior studies (e.g., Ng et al., 2019), research examining the indirect pathways through specific parenting practices is still scarce and worth further investigation. Parental autonomy support and psychological control, two independent but highly related constructs (Soenens et al., 2009; Kunz and Grych, 2013), may serve as two variables linking the indirect pathways. As Wang et al. (2007) concluded, psychological control intrudes the sense of self-determination of children, whereas autonomy support facilitates children's individuation, both of which are related to the children's development of either emotional or academic functioning. Therefore, these two specific parenting practices may link with parental expectations toward their children to construct self and relationships with others, as well as to gain academic achievement.

Autonomy support refers to parents' support to children's development of individuality and the ability of self-determination (Wang et al., 2007). Several previous studies have provided direct evidence for the associations between parental socialization goals and autonomy support. For example, parental self-development goals were positively related to parents' autonomy support (Wang et al., 2012). Parental success-pursuing goals and filial piety goals, however, were found to negatively predict parents' autonomy granting (Richman and Mandara, 2013). According to the Self-determination Theory (SDT; Deci and Ryan, 2000), individuals have a basic inclination to act autonomously and determine for themselves. The enhanced demand for autonomy along with the cognitive maturation during adolescence may alter the pattern of parent-child communication and relationship (Branje et al., 2012). In this sensitive period, parents' support for such self-determination need is especially important for promoting their children's subjective well-being, while parents' hindrance, on the contrary, increases the risk for adaptive problems including depressive symptoms (Ahmad et al., 2013). Drawing on this theoretical framework, indeed, abundant previous research revealed the preventive effect of parents' autonomy support on children's depressive symptoms (e.g., Yu et al., 2016; Lunkenheimer et al., 2017; Chen et al., 2019). Therefore, it is possible that parents' socialization goals relate to parental autonomy support, and subsequently, children's depressive symptoms.

Psychological control refers to parents' attempts to intrude into children's psychological and emotional development (Barber, 1996). Chinese parents' achievement goals were found to be positively related to parental psychological control (Luebbe et al., 2018), while maternal collectivism goals reduced such practices (Chen-Bouck et al., 2019). Parents' emphasis on children's fulfilling family obligation and striving for success was also related to their more use of authoritarian parenting style (Chao, 2000; Li et al., 2010) and strictness (Richman and Mandara, 2013), both of which encourage parents to impose restrictions on children and discourage children from challenging parents' authorities (Chao, 1994). Such psychological controlling behaviors of parents could hinder adolescents from forming secure self-perception and further impair the positive development of their psychosocial functions (Barber, 2002; Soenens and Vansteenkiste, 2010). Accumulated empirical evidence showed that psychological control contributed to a higher level of adolescent depressive symptoms both directly and indirectly through decreased self-worth, self-esteem, and unsatisfied basic psychological needs (Garber et al., 1997; Plunkett et al., 2007; Cheah et al., 2019; Rogers et al., 2020). It could also interact with other risk factors (e.g., maternal depression) to boost the occurrence of children's depression (Brennan et al., 2003). Therefore, parents' socialization goals may relate to parental psychological control, and subsequently, children's depressive symptoms.

### The Moderating Role of Educational Stage

The relations of parents' goals to parents' autonomy support as well as psychological control, and in turn, children's psychological adjustment, may vary among adolescents in different educational stages. Secondary education in Shanghai, China is divided into middle school, which normally covers 11–14 years old youth, and high school corresponding to 15–17 years old youth. Chinese high school students may experience a higher level of academic stress compared with the younger age group, which is derived from the National College Entrance Examination (NCEE), a very competitive university entrance exam that a vast majority of students have to take when they graduate from high school (Wang et al., 2016). Consequently, their perceptions and reactions to parental goals, especially those related to academic achievement, may differ from middle school students. Moreover, few studies investigated whether parental autonomy support and psychological control could affect middle school and high school students to varying degrees. A meta-analysis concerning identity development found an increase in the number of youth who have more mature identity statuses (moratorium and achieved) and a decrease in the number of youth who have less mature identity statuses (foreclosure and diffusion) from early to late adolescence (Kroger et al., 2010). Along with this common identity-formation process, parental autonomy support may exert changing influences on children's emotional well-being. In addition, parental psychological control is stated to have a devastating impact on children's self-esteem development (Lo Cascio et al., 2016), the process of which was also found to be divergent in different stages of adolescence. Normally, youth over 15 years old are in a rapid growth phase of self-esteem, while those from age 11 to 15 years are in a stagnate phase (Orth et al., 2018). Therefore, high school students may be more sensitive to the threat for self-esteem when they experience parental psychological control, which subsequently contributes to adolescent depression (Sowislo and Orth, 2013). Taken together, it is important to examine whether the relations of parental socialization goals to parental autonomy support as well as psychological control, and in turn, children's depressive symptoms vary across these two age groups of adolescents.

### The Present Study

In two cross-sectional studies using two different community samples recruited from one middle school and one high school in Shanghai, China, the current research aimed to investigate four research questions. Specifically, Study 1 investigated whether discrepancies existed between parent-reported and child-reported parental socialization goals, and whether they exhibited differential associations with adolescent depressive symptoms. Based on prior theories and empirical evidence, we hypothesized that there were discrepancies in Chinese adolescents' and their parents' perceptions in parental socialization goals (H1), and child-report was related stronger with their own depressive symptoms than parent-report (H2). Study 2 further investigated the moderated indirect effects of the associations examined in Study 1. We hypothesized that parental socialization goals were related to parental autonomy support as well as psychological control, which were associated with adolescent depressive symptoms (H3), and that such indirect effects would be different for adolescents in middle school versus high school (H4).

## Study 1

Using a multisource cross-sectional design, Study 1 examined the discrepancies in adolescents' versus their parents' perceptions of three types of parental socialization goals (i.e., self-development goals, achievement-oriented goals, and interdependence-oriented goals), and their associations with adolescent depressive symptoms among a Chinese sample.

### Materials and Methods

#### Participants and Procedures

Participants were 345 adolescent-parent dyads recruited from one middle school serving working- and middle-class families in Shanghai, China. The adolescent sample consisted of 154 (45%) boys and 191 (55%) girls. The mean age of adolescents was 12.86 years (*SD* = 0.87, range = 11–15). Most adolescents (99%) were of ethnic Han. The parent sample consisted of 80 (23%) fathers and 265 (77%) mothers. The mean age of parents was 41.71 years (*SD* = 3.17, range = 32–55). Of parents, 38% had an education lower than a college degree and 62% had a college degree or above. The majority of participants (95%) were from families with married parents and from single-child families (87%).

The research procedures were approved by the Institutional Review Board of the School of Social Development and Public Policy at Fudan University. Before the investigation, all information required for informed consent was provided to all target participants (i.e., enrolled students and their primary caregivers from grade 6 to 8) through class meetings or parent-teacher meetings. All participants were informed that participation in this study was fully voluntary and they were free to withdraw anytime. Data from students were collected by well-trained psychology graduate students through an online questionnaire in computer classrooms during school time. Parent questionnaire was completed by parents who self-identified as the primary caregivers of the participating adolescents. Both adolescents and their parents reported on parental socialization goals, and adolescents additionally reported on their depressive symptoms. The online questionnaires were used that the participants could not skip any item before the submission, so there was no missing data. An opt-out consent procedure was followed that both adolescents and parents had the opportunity to opt-out of participating in the study, and allowing their own and/or their parents' or children's data to be used for the subsequent analyses. Only adolescent-parent dyads who completed both adolescent and parent questionnaires (76% of the target participants) were included in the final sample for analysis.

#### Measures

##### Parental Socialization Goals

Adolescents and their parents reported on parental socialization goals with an 18-item scale revised by Luebbe et al. (2018) consisting of three dimensions: self-development goals (e.g., “I want my child to be good at exploring and adventuring” for parents, and “My parents want me to be good at exploring and adventuring” for children, similar revised items for child version are not presented hereinafter), achievement-oriented goals (e.g., “I want my child to achieve academic success”), and interdependence-oriented goals (e.g., “I want my child to have harmonious relationships with people around him/her”), and utilizing a five-point Likert scale ranging from 1 (*not at all desire*) to 5 (*very much desire*). All items have been used among Chinese samples in previous research (Chao, 2000; Li et al., 2010; Luebbe et al., 2018). In the current study, standard translation and back-translation procedures were followed to generate the Chinese version of this scale and ensure linguistic equivalency (Erkut, 2010).

##### Adolescent Depressive Symptoms

Adolescents completed the Chinese version of the nine-item Patient Health Questionnaire depression scale (PHQ-9; Wang et al., 2014) to report their depressive symptoms. It consists of nine items (e.g., “Little interest or pleasure in doing things”, α = 0.86) and utilizes a four-point Likert scale ranging from 0 *(not at all*) to 3 (*nearly every day*). Adolescents selected scores according to the frequencies of the symptoms described in each item over the past 2 weeks. The item scores were summed up so that higher total scores indicated more depressive symptoms. PHQ-9 has been examined to have good reliability and validity in a community sample of Chinese adolescents (Tsai et al., 2014).

#### Statistical Analysis

Data analyses were performed using SPSS 25.0 and Mplus 8.1. First, we examined the measurement model of parental socialization goals in line with Luebbe et al.'s (2018) work. Given the conceptual overlapping in items, an exploratory structural equation modeling (ESEM; Asparouhov and Muthén, 2009) framework was used to validate the three-factor structure and conduct subsequent analyses. ESEM allows factor loadings to be free estimated for non-primary factors and enables such measurement models to be integrated into the structural model. Compared with traditional CFA approaches, ESEM has superiority in fitting the data better, avoiding the distortions in structural relations caused by the highly restrictive independent cluster model (ICM), owning greater flexibility and applicability in different situations, and benefiting the subsequent multigroup invariance test (Marsh et al., 2009). The acceptable fit indices of all ESEM models are CFI >0.90, RMSEA <0.08, SRMR <0.06 (Hu and Bentler, 1999). Descriptive statistics and correlation analyses were then carried out among all the three factors of parental socialization goals reported by adolescents and their parents as well as adolescent depressive symptoms. We finally examined the associations between parent-adolescent perceived parental socialization goals as well as their discrepancies and adolescent depressive symptoms with both an ESEM model and a set of regression models.

### Results

#### The Measurement Model

**Figure 1:**
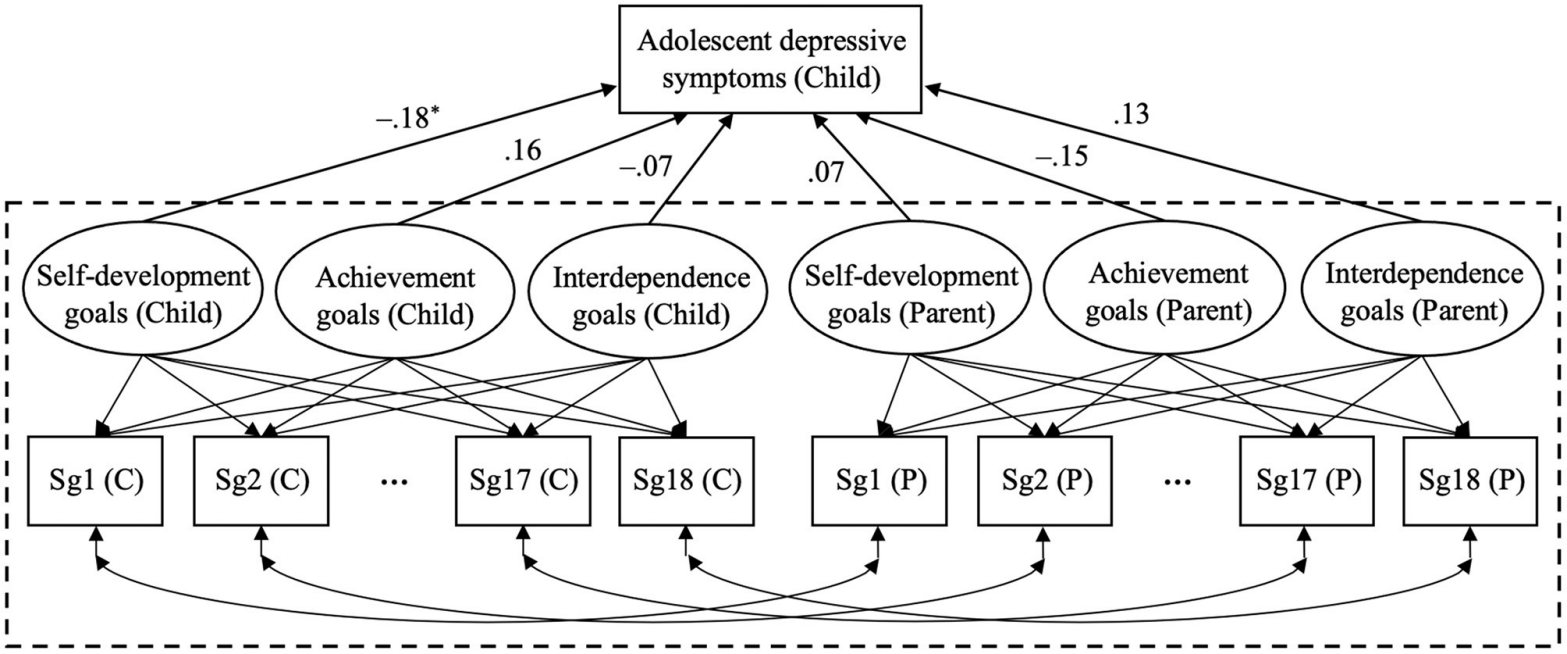
Associations between children's and parents' perceived parental socialization goals and adolescent depressive symptoms in Study 1. The three-factor ESEM model used as the measurement model is shown in the dotted box. “Sg1 (C)” to “Sg18 (C)” represent the 18 observed items reported by children. “Self-development goals (Child),” “Achievement goals (Child),” and “Interdependence goals (Child)” represent the three latent factors for children. Correspondingly, items and factors with *Parent* or *P* in parenthesis represent parents' perception. “…” represents Sg3 to Sg18 omitted for concision reason. Six latent factors are correlated though not shown in the figure. Standardized path coefficients are presented. *p < 0.05.

A three-factor repeated measurement ESEM framework (Ex.5-26; Muthén and Muthén, 2017), as shown in the part enclosed by the dotted line in Figure 1, was fit as the measurement model integrating both parents' and children's reports of parental socialization goals. The left 18 items and three factors with “Child” or “C” in parenthesis composed the first set of ESEM framework regarding children's reports (child-set), and the right 18 items and three factors with “Parent” or “P” in parenthesis composed the second set derived from parents' reports (parent-set). The residual of each item in the child-set was allowed to covary with each respective item in the parent-set, for example, Sg1 (C) with Sg1 (P). The latent constructs were correlated. The maximum likelihood estimation with robust standard errors (i.e., MLR in Mplus), which is an estimator robust to non-normality and non-independence of observations (Muthén and Muthén, 2017), and GEOMIN rotation were used for estimation.

To ensure the effectiveness of the later comparisons between informants based on the measurement model, we first tested the measurement invariance by comparing the following sequence of models—the baseline model (i.e., unconstrained model) that allowed all parameters to be freely estimated, the metric invariance model that additionally constrained the factor loadings in the two sets (i.e., child-set and parent-set) to be equal, and the scalar invariance model that further constrained the intercepts of items in the two sets to equality. Models were compared with the alternative fit indices (AFIs; Cheung and Rensvold, 2002; Chen, 2007; Meade et al., 2008), according to which, changes within 0.010 for CFI, 0.015 for RMSEA, and 0.030 for SRMR suggest metric invariance, whereas changes within 0.010 for CFI, 0.015 for RMSEA, and 0.010 for SRMR suggest scalar invariance. The results showed that the metric model fit the data acceptably, $\chi_{\left(546\right)}^{2}$ = 1111.80, *p* < 0.001, CFI = 0.90, TLI = 0.88, RMSEA = 0.06, SRMR = 0.06, and did not have notably worse model fit than the baseline model, $\chi_{\left(501\right)}^{2}$ = 1050.54, *p* < 0.001, CFI = 0.90, TLI = 0.87, RMSEA = 0.06, SRMR = 0.04; ${\Delta \text{S-B}}_{{\text{χ}}^{2}}$_(45)_ = 70.06 (Satorra and Bentler, 2010), *p* = 0.01, ΔCFI = −0.003, ΔRMSEA = −0.001, ΔSRMR = 0.015. The scalar invariance was also established with the scalar model, $\chi_{\left(561\right)}^{2}$ = 1187.70, *p* < 0.001, CFI = 0.89, TLI = 0.87, RMSEA = 0.06, SRMR = 0.06, not worsening the model fit notably than the metric model, ${\Delta \text{S-B}}_{{\text{χ}}^{2}}$_(15)_ = 69.37, *p* < 0.001, ΔCFI = −0.010, ΔRMSEA = 0.002, ΔSRMR = 0.004. The specific factor loadings, which were constrained to equality for child-report and parent-report in the final measurement model, are presented in the Supplementary Table 1.

#### Descriptive Statistics and Correlation Analysis

Results of descriptive statistics and correlation analysis are displayed in Table 1. Three factors of parental socialization goals from children's perceptions were significantly correlated with each other (0.30 < *r*s <0.71, *p*s <0.005), and the same pattern was shown in parents' perceptions (0.32 < *r*s <0.57, *p*s <0.001). Among the three types of goals, only achievement-oriented goals reported by children and parents were significantly correlated with each other (*r* = 0.12, *p* = 0.05). No significant correlation existed between children's and parents' reports regarding the other two types of goals.

**Table 1:** Correlations, means, and standard deviations of all variables.

	**1**	**2**	**3**	**4**	**5**	**6**	**7**
1. Self-development goals (Child)	–						
2. Achievement goals (Child)	0.30^**^	–					
3. Interdependence goals (Child)	0.46^***^	0.71^***^	–				
4. Self-development goals (Parent)	0.03	−0.05	−0.01	–			
5. Achievement goals (Parent)	0.03	0.12^*^	−0.01	0.32^**^	–		
6. Interdependence goals (Parent)	0.08	0.03	0.04	0.44^***^	0.57^***^	–	
7. Adolescent depressive symptoms (Child)	−0.17^*^	0.02	−0.06	0.07	−0.04	0.06	–
*M*	0.00	0.00	0.00	−0.54	−0.10	−0.91	5.22
*SD*	1.00	1.00	1.00	0.61	0.75	0.73	4.58

*N = 345. “Child” in parenthesis represents variables derived from child-report. “Parent” in parenthesis represents variables derived from parent-report*.

*This correlation matrix is based on the output of the exploratory structural equation modeling in Mplus*.

* *p < 0.05*,

** *p < 0.01*,

*** *p < 0.001*.

To test the mean differences in child-reported and parent-reported socialization goals, following Luebbe et al.'s (2018) approach, we compared two models in the same line with above model invariance tests. The constrained model, in which factor means were constrained to be equal in child-set and parent-set, $\chi_{\left(564\right)}^{2}$ = 1341.67, *p* < 0.001, CFI = 0.86, TLI = 0.84, RMSEA = 0.06, SRMR = 0.09, fit worse than the unconstrained model allowing factor means to vary across sets, $\chi_{\left(561\right)}^{2}$ = 1187.70, *p* < 0.001, CFI = 0.89, TLI = 0.87, RMSEA = 0.06, SRMR = 0.06; ${\Delta \text{S-B}}_{{\text{χ}}^{2}}$_(3)_ = 224.15, *p* < 0.001, ΔCFI = −0.028, ΔRMSEA = 0.006, ΔSRMR = 0.030. This indicated that significant differences in mean values of parental goals existed across informants. To further examine specific parental goals, we looked at the unconstrained model, in which means were fixed to zero in the child-set and allowed to be freely estimated in the parent-set. The standard errors of means in the parent-set could provide a significance test of their differences from zero (i.e., the child-set). As a result, parents' perceived self-development goals and interdependence-oriented goals were significantly lower than their counterparts in children's perceptions (self-development goals: Δ*M* = −0.54, *p* = 0.01; interdependence-oriented goals: Δ*M* = −0.91, *p* < 0.001), but no difference was shown in achievement-oriented goals between parent-report and child-report (Δ*M* = −0.10, *p* = 0.29).

#### Differential Associations Between Parents' versus Children's Perceived Parental Socialization Goals and Adolescent Depressive Symptoms

We examined the associations between parent-reported as well as child-reported parental socialization goals and adolescent depressive symptoms using the ESEM model shown in Figure 1. Child and parent sex and age were included as covariates, but only child age was finally retained due to its significant association with depressive symptoms. The final model fit the data acceptably: $\chi_{\left(612\right)}^{2}$ = 1218.95, *p* < 0.001, CFI = 0.89, TLI = 0.88, RMSEA = 0.05, SRMR = 0.06. The results showed that among the three types of parental goals, only children's perceived self-development goals were negatively associated with adolescent depressive symptoms, β = −0.18, *p* = 0.04. None of the three types of parent-reported goals was related to adolescent depressive symptoms.

Next, we examined how discrepancies between parents' and children's perceptions of parental socialization goals related to adolescent depressive symptoms by using the interaction terms of reports from both as predictors. A significant interaction effect indicates that the perceptual discrepancy has an effect on the dependent variable (Laird and De Los Reyes, 2013; Dimler et al., 2017). Unfortunately, it is not allowed to build the interaction term of latent variables directly in an ESEM model. Instead, we used the factor scores generated for the six latent variables of children's and parents' perceived parental goals, and further tested their interaction effects with multiple regression analysis (Distefano et al., 2008). The default estimation method for generating factor scores in Mplus is the Regression Method (Muthén and Muthén, 2017). For each type of parental goals, we set up a regression model, in which children's reports (Child), parents' reports (Parent), and their interaction term (Child × Parent) were regressors to adolescent depressive symptoms. Child age was added to the model as a covariate consistent with the ESEM model. The results are as Table 2 shows, the regression coefficients of the interaction term “Child × Parent” in all the three models were not significant, suggesting that parent-child perceptual discrepancies in parental goals were not related to adolescent depressive symptoms.

**Table 2:** Regression coefficients of the three types of parental socialization goals on adolescent depressive symptoms.

**Variable**	**Self-development goals**		**Achievement goals**		**Interdependence goals**	
	**β**	***SE***	**β**	***SE***	**β**	***SE***
Constant	0.07	0.05	0.07	0.05	0.07	0.05
Age	0.12^**^	0.05	0.14^**^	0.06	0.13^**^	0.06
Child	−0.13^**^	0.05	0.02	0.05	−0.06	0.08
Parent	0.06	0.05	−0.03	0.05	−0.03	0.05
Child × Parent	0.01	0.10	0.05	0.09	−0.05	0.05
*R*^2^	0.03		0.01		0.01	

*Three regression models are presented regarding self-development goals, achievement-oriented goals, and interdependence-oriented goals, respectively. “Child” represents parental goals reported by children. “Parent” represents parental goals reported by parents. “Child × Parent” represents the interaction term of child-report and parent-report. Child age was included as a covariate. Standardized regression coefficients are shown*.

** *p <0.01*.

### Discussion

In Study 1, hypothesis 1 was partially verified—discrepancies in children's and their parents' perceptions existed in parental self-development and interdependence-oriented goals, while not in achievement-oriented goals. Hypothesis 2 was also partly confirmed. Among the three types of goals reported by parents and children as well as their perceptual discrepancies, only adolescents' perceived self-development goals were negatively associated with their depressive symptoms.

## Study 2

Study 1 suggested that Chinese adolescents and their parents shared the same three-factor structure of parental socialization goals in their cognition but have discrepancies in the perceptions of different types of goals, among which only the association of children's self-perceived parental goals and their depressive symptoms was significant. Drawing on these findings, we further investigated the indirect associations between child-perceived parental goals and their depressive symptoms through child-perceived parental autonomy support and psychological control, and examined whether these associations varied across adolescents in different educational stages.

### Materials and Methods

#### Participants and Procedures

Participants were 424 middle school students (grade 6–8) and 301 high school students (grade 10–11) recruited from one middle school and one high school serving working- and middle-class families in Shanghai, China. The middle school sample consisted of 196 (46%) boys and 228 (54%) girls, with a mean age of 12.76 years (*SD* = 0.91, range = 10–14). The majority of them were of ethnic Han (97%), from families with married parents (97%), and from single-child families (86%). Of their parents, 28% had an education lower than a college degree and 72% had a college degree or above. The high school sample consisted of 140 (47%) boys and 161 (53%) girls. The mean age was 16.24 years (*SD* = 1.17, range = 14–18). The majority of them were of ethnic Han (98%), from families with married parents (90%), and from single-child families (90%). Of their parents, 52% had an education lower than a college degree and 48% had a college degree or above. The consent acquisition and data collection procedures were consistent with Study 1. Participants reported on parental socialization goals, autonomy support, psychological control, and their own depressive symptoms through a questionnaire voluntarily.

#### Measures

##### Parental Socialization Goals and Adolescent Depressive Symptoms

Child self-perceived parental socialization goals and depressive symptoms were measured using the same scales in Study 1 (α = 0.88 for PHQ-9).

##### Parental Autonomy Support

The Chinese version of an eight-item parental autonomy support scale (McPartland and Epstein, 1977; Steinberg et al., 1992) revised by Wang et al. (2007) was used to measure adolescent self-perceived parental autonomy support (e.g., “My parents allow me to make choices about my own things”; α = 0.95). The scale utilizes a five-point Likert scale ranging from 1 (*not at all true*) to 5 (*completely true*). The item scores were averaged, such that higher average scores indicated higher levels of adolescents' perceived autonomy support from their parents.

##### Parental Psychological Control

The Chinese version of a ten-item parental psychological control scale (Barber, 1996; Silk et al., 2003) revised by Wang et al. (2007) was used to measure adolescent self-perceived parental psychological control (e.g., “My parents tell me that I should feel guilty when I do not do as well as they expect me to”; α = 0.91). Adolescents responded to the items using a five-point Likert scale ranging from 1 (*not at all true*) to 5 (*completely true*). The higher average scores indicated higher levels of adolescents' perceived parental psychological control.

#### Statistical Analysis

Data analyses were performed using SPSS 25.0 and Mplus 8.1. We first examined the measurement model and its multigroup invariance with an ESEM approach. Then, we conducted descriptive statistics and correlation analyses among all study variables by educational stage. Finally, we investigated the indirect effect model including parental autonomy support and psychological control, and examined whether these associations differed among middle versus high school students using a multigroup analysis.

### Results

#### The Measurement Model

A standard three-factor ESEM framework was adopted to validate the latent structure of parental socialization goals in the new sample. Again, for estimation, we used MLR estimator and GEOMIN rotation. The measurement model was first fit in the total sample and the two separate groups of middle school and high school students. The fit indices of all models were acceptable, χ^2^s_(102)_ <322.74, *p*s <0.001, CFIs > 0.93, TLIs > 0.89, RMSEAs <0.06, SRMRs <0.04. Then, we compared three models to test the measurement invariance. The baseline model allowed all parameters to vary across groups. The metric invariance model that additionally constrained factor loadings to be equal in the two groups showed a good model fit, $\chi_{\left(249\right)}^{2}$ = 482.78, *p* < 0.001, CFI = 0.94, TLI = 0.93, RMSEA = 0.05, SRMR = 0.05, and did not fit notably worse than the baseline model, $\chi_{\left(204\right)}^{2}$ = 409.30, *p* < 0.001, CFI = 0.95, TLI = 0.93, RMSEA = 0.05, SRMR = 0.03; ${\Delta \text{S-B}}_{{\text{χ}}^{2}}$_(45)_ = 73.24, *p* = 0.005, ΔCFI = −0.006, ΔRMSEA = −0.002, ΔSRMR = 0.018. The scalar invariance model that further constrained intercepts of items to equality fit the data good, $\chi_{\left(264\right)}^{2}$ = 539.19, *p* < 0.001, CFI = 0.93, TLI = 0.92, RMSEA = 0.05, SRMR = 0.06, and did not have notably worse model fit than the metric model, ${\Delta \text{S-B}}_{{\text{χ}}^{2}}$_(15)_ = 59.70, *p* < 0.001, ΔCFI = −0.010, ΔRMSEA = 0.003, ΔSRMR = 0.009, supporting the scalar invariance among the middle school and high school students.

#### Descriptive Statistics and Correlation Analysis

Results of descriptive statistics and correlation analysis by educational stage are presented in Table 3. High school students had significantly lower self-perceived parental autonomy support (Δ*M* = −0.58, *p* < 0.001) and higher depressive symptoms (Δ*M* = 3.26, *p* < 0.001) than middle school students. Mean comparisons of three latent factors of parental goals were conducted with the similar method as Study 1. We compared a constrained model that constrained means to be equal in two groups with an unconstrained model that allowed means to vary across groups. Factor mean invariance was rejected as the constrained model, $\chi_{\left(267\right)}^{2}$ = 678.16, *p* < 0.001, CFI = 0.90, TLI = 0.89, RMSEA = 0.07, SRMR = 0.13, notably worsened the model fit compared with the unconstrained model, $\chi_{\left(264\right)}^{2}$ = 539.19, *p* < 0.001, CFI = 0.93, TLI = 0.92, RMSEA = 0.05, SRMR = 0.06; ${\Delta \text{S-B}}_{{\text{χ}}^{2}}$_(3)_ = 349.21, *p* < 0.001, ΔCFI = −0.033, ΔRMSEA = 0.011, ΔSRMR = 0.070. The results from the unconstrained model further showed that high school students' perceived self-development goals and achievement-oriented goals were significantly lower than middle school students (self-development goals: Δ*M* = −1.10, *p* < 0.001; achievement-oriented goals: Δ*M* = −0.47, *p* < 0.001).

**Table 3:** Correlations, means, and standard deviations of all variables by educational stage.

	**1**	**2**	**3**	**4**	**5**	**6**
1. Self-development goals	–	−0.05	0.39^**^	0.60^***^	−0.35^**^	−0.24^**^
2. Achievement-oriented goals	0.50^***^	–	0.38^**^	−0.30^***^	0.47^***^	−0.01
3. Interdependence-oriented goals	0.48^***^	0.52^***^	–	0.14	0.15^*^	−0.18^**^
4. Autonomy support	0.46^***^	−0.03	0.16^*^	–	−0.58^***^	−0.26^***^
5. Psychological control	0.05	0.39^***^	0.12^*^	−0.33^***^	–	0.26^***^
6. Adolescent depressive symptoms	−0.18^**^	0.09	−0.09	−0.37^***^	0.25^***^	–
**Middle school (*****n*** **= 424)**						
*M*	0.00	0.00	0.00	3.82	3.00	4.48
*SD*	1.00	1.00	1.00	1.08	1.09	4.62
**High school (*****n*** **= 301)**						
*M*	−1.10	−0.47	−0.13	3.24	2.91	7.74
*SD*	0.94	1.04	0.94	0.96	1.02	5.05
*t*	–	–	–	7.51^***^	1.11	−9.03^***^
Cohen's *d*	–	–	–	0.57	0.09	0.67

*All variables were reported by children. Correlations below the diagonal are for students from middle school. Correlations above the diagonal are for students from high school. Mean difference tests of the three factors are shown in text. This correlation matrix is based on the output of the exploratory structural equation modeling in Mplus*.

* *p <0.05*,

** *p <0.01*,

*** *p <0.001*.

#### Multigroup Analysis of the Indirect Effect Model

**Figure 2:**
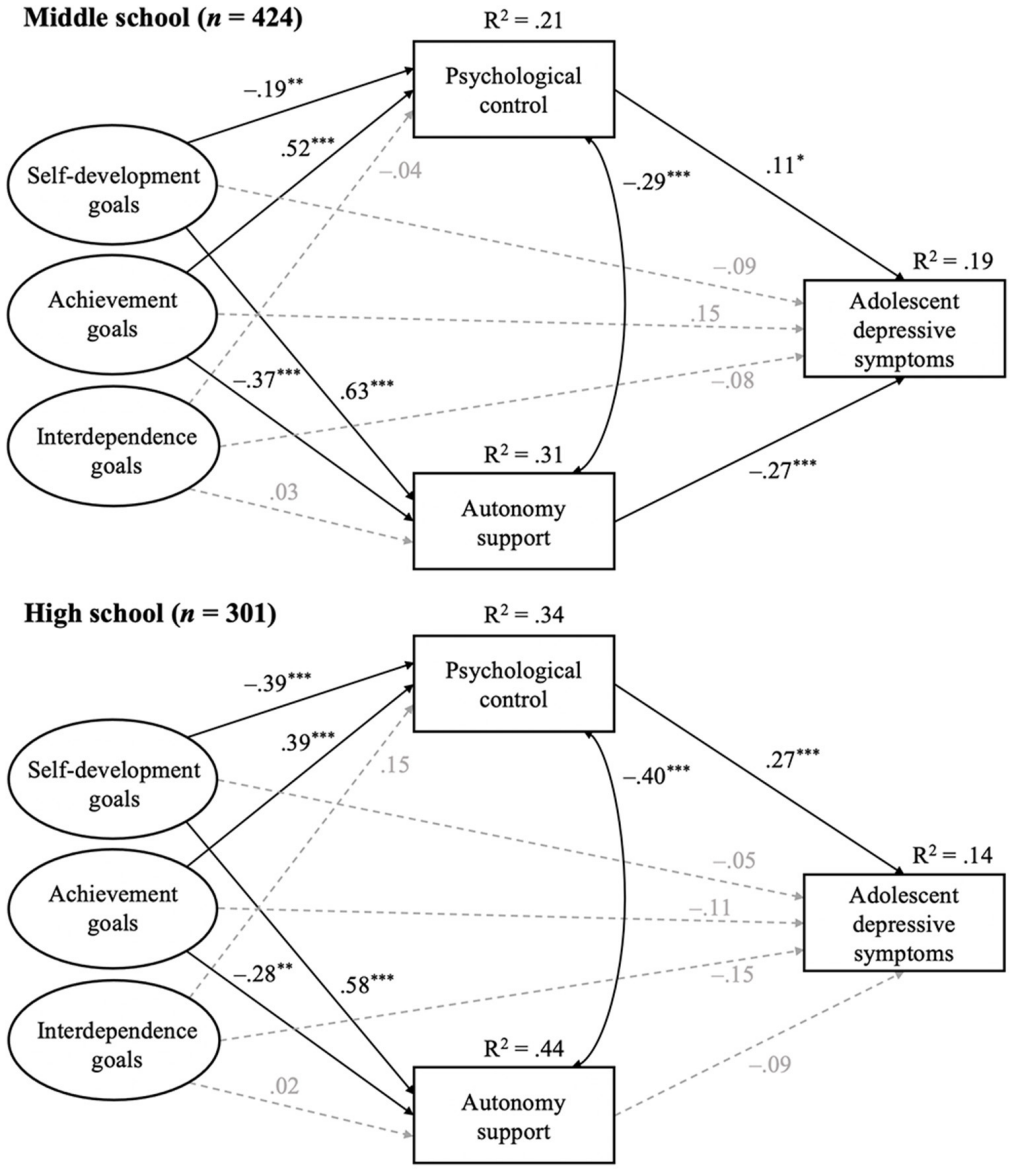
Multigroup indirect effect model of adolescents' self-perceived parental socialization goals, autonomy support, psychological control, and adolescent depressive symptoms in Study 2. **Top** panel shows the structural model for middle school students. **Bottom** panel shows the structural model for high school students. All variables were reported by children. Standardized path coefficients are presented. *p < 0.05, **p < 0.01, ***p < 0.001.

We ran a multigroup ESEM to examine the indirect pathways from the three types of self-perceived parental socialization goals to adolescent depressive symptoms through parental autonomy support and psychological control (Figure 2). Child age was included as a covariate consistent with Study 1. The residuals of autonomy support and psychological control were allowed to covary with each other. All structural paths were free estimated in separate groups. The results showed a good model fit, $\chi_{\left(369\right)}^{2}$ = 666.83, *p* < 0.001, CFI = 0.94, TLI = 0.93, RMSEA = 0.05, SRMR = 0.05.

The total, direct and indirect effects in each model were estimated using the Boostrap confidence interval with 1000 resamples (Preacher and Hayes, 2008). Although it is not possible to employ the Bootstrap technique in an ESEM model, the newly developed ESEM-Within-CFA approach (EwC) allowed us to circumvent this limitation by transforming the ESEM solution into a CFA framework by setting up starting values (Marsh et al., 2014). The results are shown in Table 4. Indirect paths from self-development goals as well as achievement-oriented goals to parental autonomy support to adolescent depressive symptoms were significant in the middle school sample, whereas indirect paths from self-development goals as well as achievement-oriented goals to parental psychological control to adolescent depressive symptoms were significant in the high school sample. Specifically, self-development goals were positively, whereas achievement-oriented goals were negatively, related to autonomy support, which was negatively associated with adolescent depressive symptoms. Psychological control, in contrast, was related to all other variables in the opposite directions to autonomy support.

**Table 4:** Total, direct, and indirect effects of self-development goals and achievement-oriented goals on adolescent depressive symptoms.

	**Estimate**	**95% CI**
**Middle school (*****n*** **=** **424)**		
Total effect: self-development goals—depressive symptoms	−0.28^***^	[−0.44, −0.12]
Direct effect	−0.09	[−0.27, 0.11]
Indirect effect: self-development goals—autonomy support—depressive symptoms	−0.17^***^	[−0.28, −0.08]
Indirect effect: self-development goals—psychological control—depressive symptoms	−0.02	[−0.05, 0.00]
Total effect: achievement goals—depressive symptoms	0.30^***^	[0.15, 0.44]
Direct effect	0.15	[−0.04, 0.31]
Indirect effect: achievement goals—autonomy support—depressive symptoms	0.10^**^	[0.04, 0.17]
Indirect effect: achievement goals—psychological control—depressive symptoms	0.06	[−0.00, 0.12]
**High school (*****n*** **=** **301)**		
Total effect: self-development goals—depressive symptoms	−0.20^*^	[−0.39, −0.00]
Direct effect	−0.05	[−0.28, 0.21]
Indirect effect: self-development goals—autonomy support—depressive symptoms	−0.05	[−0.18, 0.06]
Indirect effect: self-development goals—psychological control—depressive symptoms	−0.11^**^	[−0.19, −0.04]
Total effect: achievement goals—depressive symptoms	0.02	[−0.16, 0.21]
Direct effect	−0.11	[−0.30, 0.11]
Indirect effect: achievement goals—autonomy support—depressive symptoms	0.02	[−0.03, 0.09]
Indirect effect: achievement goals—psychological control—depressive symptoms	0.11^**^	[0.04, 0.19]

*All variables were reported by children. Standardized estimates are shown*.

* *p <0.05*,

** *p <0.01*,

*** *p <0.001*.

We also ran the reversed causal model as an alternate model due to our cross-sectional design. That is, the reversed model used the same variables, estimation methods, and invariance constraints as the hypothesized model, with only the direction of the pathways between variables being reversed (i.e., from adolescent depressive symptoms to parental autonomy support and psychological control, then to the three types of parental socialization goals). This reversed multigroup ESEM fit the data good, $\chi_{\left(369\right)}^{2}$ = 666.81, *p* < 0.001, CFI = 0.94, TLI = 0.93, RMSEA = 0.05, SRMR = 0.05, and revealed several significant indirect pathways, for example, the pathway that adolescent depressive symptoms were negatively related to parental autonomy support, which was positively related to parental self-development goals (full results available on request). The results suggested the possible reciprocal relationships between the study variables.

### Discussion

In Study 2, the indirect pathways from parental socialization goals to adolescent depressive symptoms through parental autonomy support and psychological control proposed in hypothesis 3 was verified. Hypothesis 4 was also supported by the different patterns of the above links shown in middle school and high school students. Notably, a significant total effect from parental achievement-oriented goals to adolescent depressive symptoms among middle school students was shown in Study 2, which was inconsistent with the results from Study 1. Study 1 estimated this path coefficient with a relatively small sample size (*N* = 345) and a more complicated repeated measurement model that included both parent-report and child-report, and we observed an estimate with a trend to be significant (β = 0.16, *p* = 0.14). Therefore, we tend to conclude that there was a significant but small effect of parental achievement-oriented goals on adolescent depressive symptoms among middle school students, which emerged in Study 2 with a bigger sample size (*N* = 424) and a simplified model.

## General Discussion

Parents' beliefs and goals toward their children may guide their parenting practices, which in turn exert influences on children's developmental outcomes (Darling and Steinberg, 1993; Bornstein et al., 2017). The current study yielded new evidence for this integrative model of parenting in a Chinese sample. Given that the results indicated that only children's but not parents' perceived parental socialization goals were associated with children's depressive symptoms (Study 1), we further examined how children's perceived parental autonomy support and psychological control linked this significant relation in different educational stages (Study 2). Specifically, the indirect effects of self-development goals and achievement-oriented goals on depressive symptoms through parental autonomy support were significant among middle school students, whereas the indirect effects through parental psychological control were present among high school students.

Guided by the three-factor structure of parental socialization goals proposed by Luebbe et al. (2018), we first observed the differences in parents' and children's reports on this construct. Parent-child perceptual discrepancies have been examined in previous research on different dimensions of parenting (e.g., monitoring behaviors; Augenstein et al., 2016; warmth and negativity; Feinberg et al., 2000), but not on parental goals. Results of Study 1 showed a significant, albeit weak, correlation between parent- and adolescent-reports on achievement-oriented goals, but not on self-development goals or interdependence-oriented goals, both of which showed significantly higher children's reports than parents' reports. A possible reason for the divergence between different types of parental goals is that the parent-child communication regarding different goals may vary. Chinese parents were found to place a strong emphasis on goals of academic achievement and family obligation compared to Western parents (Pearson and Rao, 2003; Qu et al., 2016). Moreover, Chinese youth often spend most of their time in academic-related activities such as taking school curriculum, taking extracurricular classes, and doing homework (Sun et al., 2012). Therefore, dialogues about academics between Chinese youth and their parents may appear far more frequently in their daily life compared with topics regarding self-development and interdependence. Such frequent opinion exchange may lead to relatively higher consistency in parent-adolescent perceptions in parental achievement-oriented goals. With regard to the other two types of goals, when children were asked to report but did not actually know their parents' ideas, they may eventually make a guess based on the expectations of their own or of the social values. Specifically, adolescents may sometimes overestimate parents' expectations on their development of independence to maintain an illusion of autonomy (Cheung et al., 2016). They may also overrate parents' interdependence-oriented goals after being cultivated under the Chinese culture that is relatively more collectivistic and less individualistic (Oyserman et al., 2002). In other words, their reports may represent the expectations of the values and norms of an entire collectivistic society for its individuals' development.

Study 1 also suggested that only children's perceived parental socialization goals were significantly associated with their depressive symptoms, an effect not present for either parents' reports or child-parent perceptual discrepancies. Prior work has shown that adolescents' reports only had low-to-moderate magnitudes of associations with reports from other informants (e.g., parent and researcher) when assessing parenting (Gonzales et al., 1996; Laird and De Los Reyes, 2013). However, compared with parenting practices reported by others, what are actually perceived by children may play a larger role in their developmental outcomes (Dimler et al., 2017). The results of Study 1 provide new evidence to this line of findings with a focus on the role of parental expectations toward their children.

Study 2 further explored the moderated indirect effects of adolescents' self-perceived parental socialization goals on their depressive symptoms. Notably, the three types of goals exhibited divergent relationships with adolescent depressive symptoms. Self-development goals were negatively associated with depressive symptoms across samples and across educational stages in the two studies, suggesting it as a stable protective factor of adolescent emotional development. In contrast, achievement-oriented goals were shown to be a risk factor, which was positively related to adolescent depressive symptoms only among middle school students, but not among high school students. The impact of parenting on adolescent development could be moderated by adolescents' openness to parental socialization (Darling and Steinberg, 1993), which could be a possible reason for this difference between the two groups. With the continuous maturity of cognition and socialization, as well as the high-intensity academic pressure brought by the approaching National College Entrance Examination and motivating school atmosphere (Liu and Lu, 2012; Sun et al., 2012), youth's awareness of family responsibility and pursuit of academic achievement may grow increasingly after they enter high school (Taylor et al., 1997), which then mitigates the potential negative impact of parents' achievement-oriented goals on their emotional well-being. With regard to the interdependence-oriented goals, no association of which with adolescent depressive symptoms was found in both Study 1 and Study 2. This result was consistent with Luebbe et al.'s (2018) finding with adolescent anxiety as the outcome.

We then identified two indirect pathways from adolescents' perceived parental self-development goals as well as achievement-oriented goals to their depressive symptoms through adolescents' perceived parental autonomy support and psychological control. More endorsement of self-development goals was related to increased autonomy support and decreased psychological control, both of which were related to fewer adolescent depressive symptoms. It is possible that parents who encourage their children to develop individuality and independence are more likely to give their children more freedom to self-explore and self-express, and prefer to play a role of guider rather than authority, which is characterized as high autonomy support and low psychological control. Such parenting practices provide a proper environment for children to develop autonomy and thereby decrease the risk of depressive symptoms (Giessen et al., 2014). With regard to the link between achievement-oriented goals and adolescent depressive symptoms, the two indirect paths through autonomy support and psychological control were also found to be significant. That is, parents who expect their children to achieve success and bring honors to the family may tend to use excessively intrusive parenting practices in an effort to achieve such goals (Luebbe et al., 2018). The pressure experienced by parents to make their children perform well may also easily translate into high-control and low-autonomy parenting behaviors (Grolnick et al., 2002), which could be increasingly detrimental to youth's emotional development in the contemporary China that has been integrating Western individualistic culture emphasizing on independence and uniqueness in the process of globalization (Lamm et al., 2008).

Finally, the significance of the indirect paths from parental goals to adolescent depressive symptoms through autonomy support and psychological control was shown to be divergent in different educational stages—indirect paths through autonomy support were evident among middle school students, whereas indirect paths through psychological control were evident among high school students. From early to late adolescence, youth demonstrate a decrease in their attachment to parents (Nickerson and Nagle, 2005), and a gradually forming autonomy and identity (McElhaney et al., 2009). As they navigate the adolescent years, youth also tend to seek emotional support more from friends and romantic partners instead of their parents (Markiewicz et al., 2006). Therefore, parents' autonomy support may have a more salient influence among middle school students who are in their early adolescence. In contrast, high school students are stepping into a rapid developmental stage of self-esteem after a lag phase from ages 11 to 15 (Orth et al., 2018). The overly-intrusive and emotionally-manipulative psychological control strategies, such as guilt induction and love withdrawal used by parents, could have a destructive impact on youth's growing self-esteem and subsequently contribute to adolescent depressive symptoms (Sowislo and Orth, 2013; Lo Cascio et al., 2016). This could be a possible reason why parents' psychological control played a more salient role in the links between parental goals and adolescent depressive symptoms among mid-to-late adolescents.

### Limitations and Implications

The current study has some limitations that point to directions for future research. First, the correlational nature of the cross-sectional data does not allow us to make a causal inference and limit us to verify the theoretical mediation model. Based on the current pilot investigation, future longitudinal approaches are needed to examine the long-term effects of parental socialization goals on parenting practices and adolescent depressive symptoms, and to fully test the possible mediating role of parenting practices in the links between parental goals and adolescent outcomes in line with the integrative model of parenting (Darling and Steinberg, 1993). Experimental work that manipulates parental goals in lab settings can explore such causal links. In addition, adolescent depressive symptoms may not only lead to children's negative perceptual and recalling biases toward parents (De Los Reyes et al., 2008), but also affect the expectations held and the parenting practices used by their parents toward them. Therefore, future research may examine the possible reversed causal links from adolescent outcomes pointing to parental behaviors and beliefs, which has been supported by the results of our preliminary exploration in the current study by running the reversed indirect effect models. Second, fathers were relatively fewer than mothers in Study 1, which may weaken the representativeness of the parent sample. Previous studies have shown that mother-child and father-child relationships can differ (Laible, 2004; Cruz et al., 2020; Ravindran et al., 2020). Future research may explore the associations between parents' goals, parenting, and children's depressive symptoms in parent-child dyads of different sex combinations (e.g., mother-son or father-son), respectively. Third, all variables were reported by children in Study 2, which may exaggerate the effects we observed that possibly derived from the common method bias. Future research may adopt more objective approaches (e.g., third-party observers) to measure parenting practices and children's depressive symptoms to further verify the proposed model. Fourth, the sample consisted of students from different classrooms, which led to a nested structure of the data used in this study (i.e., participants were nested within classrooms). However, the independence assumption was not tested, and the multilevel modeling was not considered due to the unavailability of information on classroom membership that may also contribute to adolescent depressive symptoms (Shochet and Smith, 2014). Future research may consider taking classroom membership into account when exploring the associations between parenting and adolescent depressive symptoms. Fifth, all participants were living in Shanghai, a large-size urban city in China, which may lead to relatively high homogeneity of the sample. Diversified groups of individuals from different subcultures, cities, and families of different socioeconomic statuses are worth further investigation. Future research may also consider exploring the possible reasons that cause parents' and children's perceptual discrepancies in parental socialization goals, and their effects on other dimensions of parenting (e.g., harshness, guan) and adolescents' developmental outcomes (e.g., externalizing problems, academic performance).

Despite the limitations above, the current research examined, for the first time, the parent-adolescent discrepancies in perceptions of parental socialization goals and their differential associations with adolescent depressive symptoms. The results highlight the importance of children's perceptions, instead of parents' perceptions, in relation to children's depressive symptoms. As adolescents were found to perceive their family environment and parenting behaviors more negatively compared with parents (De Los Reyes et al., 2016), it is important for parents to become aware of such possible incongruence and enhance communication with their children to bridge the gap in their perceptions. Moreover, our findings also indicated that the indirect paths through autonomy support and psychological control may work to varying degrees among middle school and high school students. Therefore, parents should pay particular attention to the use of specific parenting behaviors when children are in different educational stages.

## Data Availability Statement

The raw data supporting the conclusions of this article will be made available by the authors, without undue reservation.

## Ethics Statement

The studies involving human participants were reviewed and approved by the Institutional Review Board of the School of Social Development and Public Policy at Fudan University. Written informed consent from the participants' legal guardian/next of kin was not required to participate in this study in accordance with the national legislation and the institutional requirements.

## Author Contributions

ZZ developed the hypotheses, conducted the analyses, drafted the manuscript, and involved in data collection. ML and JW were involved in data collection and revised the manuscript. XL designed and supervised the study, helped to develop the hypotheses, and revised the manuscript. All authors read and approved the final manuscript.

## Conflict of Interest

The authors declare that the research was conducted in the absence of any commercial or financial relationships that could be construed as a potential conflict of interest.
