# Supplementary material for: Differential Associations Between Parents' Versus Children's Perceptions of Parental Socialization Goals and Chinese Adolescent Depressive Symptoms
Source: Front Psychol. 2021 Jun 25;12:681940. doi: 10.3389/fpsyg.2021.681940 (PMC8267414; doi:10.3389/fpsyg.2021.681940)
Supplement: Supplementary file 1 [file Table_1.DOCX]

Supplementary Table 1. Exploratory structural equation model factor loadings for parental socialization goals in Study 1

| Item | SD | AC | IN |
| --- | --- | --- | --- |
| I want my child to be very unique and be his/hers own individual. | **.73** | .03 | .05 |
| I want my child to become involved in non-academic activities at school. | **.80** | **–.16** | .01 |
| I want my child to have strong sense of self-respect. | **.62** | **.26** | –.04 |
| I want my child to be good at exploring and adventuring. | **.84** | –.01 | .14 |
| I want my child to honor the family. | .03 | **.77** | .02 |
| I want my child to compete with classmates. | –.01 | **.79** | –.05 |
| I want my child to be the top student. | **–.21** | **.65** | .01 |
| I want my child to be aware of other people’s expectations. | **.21** | **.58** | .12 |
| I want my child to achieve academic success. | **–.14** | **.35** | **.44** |
| When in a group, I want my child to be cooperative with people. | **.23** | .01 | **.52** |
| I want my children to respect their elders. | –.02 | **–.10** | **.64** |
| I want my child to be modest and learn from others. | **–.14** | .00 | **.68** |
| I want my child to have harmonious relationships with people around him/her. | –.01 | –.11 | **.75** |
| I want my child to be self-expressive. | .06 | –.05 | **.64** |
| When my child succeeds, I want him/her to think about the help he/she received from others. | .14 | .14 | **.44** |
| I want my child to know the role he/she should play in a social group. | .**21** | .15 | **.54** |
| I want my child to be popular amongst his/her classmates. | .06 | .16 | **.51** |
| I want my child to meet people from other cultures. | .17 | .15 | **.51** |

*Factor loadings of each item on each of the three factors in Study 1 are presented. “SD” represents the “self-development goals” factor, “AC” represents the “achievement-oriented goals” factor, “IN” represents the “interdependence-oriented goals” factor. Loadings in bold are significant at the .05 level.* *The factor loadings in Study 2 showed a similar pattern with Study 1, which are therefore not reported repeatedly.*
